# Ethyl 6-(4-chloro­phen­yl)-4-(4-fluoro­phen­yl)-2-oxo­cyclo­hex-3-ene-1-carboxyl­ate

**DOI:** 10.1107/S1600536813031851

**Published:** 2013-11-30

**Authors:** M. Sapnakumari, B. Narayana, H.S. Yathirajan, Jerry P. Jasinski, Ray J. Butcher

**Affiliations:** a Department of Studies in Chemistry, Mangalore University, Mangalagangotri 574 199, India; b Department of Studies in Chemistry, University of Mysore, Manasagangotri, Mysore 570 006, India; c Department of Chemistry, Keene State College, 229 Main Street, Keene, NH 03435-2001, USA; d Department of Chemistry, Howard University, 525 College Street NW, Washington, DC 20059, USA

## Abstract

The asymmetric unit of the title compound, C_21_H_18_ClFO_3_, contains two independent mol­ecules. In one mol­ecule (*A*), the 4-chloro­phenyl, oxo­cyclo­hex-3-ene, carboxyl­ate, and ethyl groups were refined as disordered over two sets of sites with a 0.684 (5):0.316 (5) ratio. The cyclo­hexene ring in the disordered mol­ecule is in a slightly distorted envelope conformation for the major component (with the C atom bound to the carboxylate group being the flap atom) and in a screw-boat conformation for the minor component. In the ordered mol­ecule (*B*), the cyclo­hexene ring is in a half-chair conformation. The dihedral angles between the mean planes of the fluoro- and chloro-substituted benzene rings are 89.9 (7) (only the major component is considered for *A*) and 76.4 (7)° (*B*). In the crystal, inversion dimers are observed along with weak C—H⋯O hydrogen bonds, which form chains along [100].

## Related literature

For the synthesis and applications of 4,6-diaryl-2-oxo-cyclo­hex-3-ene-1-carboxyl­ate derivatives, see: Ashalatha *et al.* (2009 ▶); Sreevidya *et al.* (2010 ▶); Padmavathi *et al.* (2000 ▶); Senguttuvan & Nagarajan (2010 ▶); Butcher *et al.* (2011 ▶). For puckering parameters, see: Cremer & Pople (1975 ▶). For standard bond lengths, see Allen *et al.* (1987 ▶). For related structures, see: Dutkiewicz *et al.* (2011*a* ▶,*b* ▶,*c* ▶); Fun *et al.* (2010 ▶); Harrison *et al.* (2010 ▶); Kant *et al.* (2012 ▶).
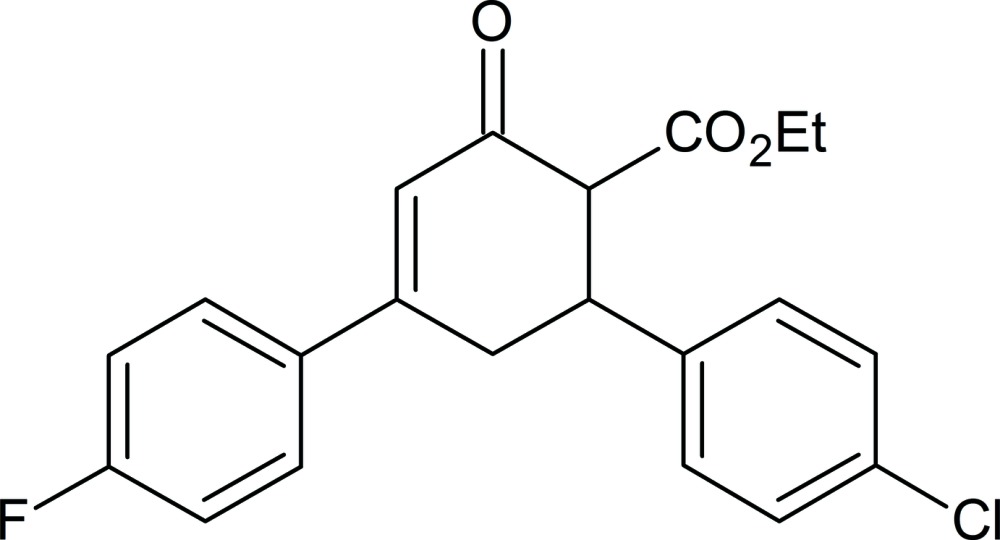


## Experimental

### 

#### Crystal data

C_21_H_18_ClFO_3_
*M* *_r_* = 372.80
Triclinic, 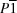

*a* = 11.6611 (5) Å
*b* = 13.1823 (5) Å
*c* = 13.2251 (5) Å
α = 77.250 (3)°
β = 87.320 (3)°
γ = 67.342 (4)°
*V* = 1828.15 (14) Å^3^
*Z* = 4
Cu *K*α radiation
μ = 2.09 mm^−1^
*T* = 123 K
0.35 × 0.23 × 0.18 mm

#### Data collection

Agilent Xcalibur (Ruby, Gemini) diffractometer
Absorption correction: multi-scan (*CrysAlis PRO* and *CrysAlis RED*; Agilent, 2012 ▶) *T*_min_ = 0.508, *T*_max_ = 1.000
13437 measured reflections
7365 independent reflections
6861 reflections with *I* > 2σ(*I*)
*R*_int_ = 0.021

#### Refinement

*R*[*F* ^2^ > 2σ(*F* ^2^)] = 0.055
*wR*(*F* ^2^) = 0.148
*S* = 1.09
7365 reflections
617 parameters
177 restraints
H-atom parameters constrained
Δρ_max_ = 0.68 e Å^−3^
Δρ_min_ = −0.40 e Å^−3^

### 

Data collection: *CrysAlis PRO* (Agilent, 2012 ▶); cell refinement: *CrysAlis PRO*; data reduction: *CrysAlis RED* (Agilent, 2012 ▶); program(s) used to solve structure: *SHELXS97* (Sheldrick, 2008 ▶); program(s) used to refine structure: *SHELXL97* (Sheldrick, 2008 ▶); molecular graphics: *SHELXTL* (Sheldrick, 2008 ▶); software used to prepare material for publication: *SHELXTL*.

## Supplementary Material

Crystal structure: contains datablock(s) I. DOI: 10.1107/S1600536813031851/lh5662sup1.cif

Structure factors: contains datablock(s) I. DOI: 10.1107/S1600536813031851/lh5662Isup2.hkl

Supplementary material file. DOI: 10.1107/S1600536813031851/lh5662Isup3.cml

Additional supplementary materials:  crystallographic information; 3D view; checkCIF report

## Figures and Tables

**Table 1:** Hydrogen-bond geometry (Å, °)

*D*—H⋯*A*	*D*—H	H⋯*A*	*D*⋯*A*	*D*—H⋯*A*
C8*A*—H8*A*⋯O1*B*	0.95	2.55	3.478 (3)	167
C2*A*—H2*AA*⋯O1*B*	0.99	2.44	3.288 (6)	143
C2*A*—H2*AB*⋯O2*B* ^i^	0.99	2.56	3.432 (7)	147
C2*C*—H2*CA*⋯O1*B*	0.99	2.32	3.278 (18)	164
C2*C*—H2*CB*⋯O2*B* ^i^	0.99	2.42	3.40 (2)	170
C2*B*—H2*BA*⋯O1*A* ^ii^	0.99	2.49	3.315 (8)	140
C2*B*—H2*BA*⋯O1*C* ^ii^	0.99	2.56	3.380 (18)	140
C2*B*—H2*BB*⋯O2*C* ^iii^	0.99	2.58	3.436 (10)	145
C8*B*—H8*B*⋯O1*A* ^ii^	0.95	2.50	3.379 (8)	154
C8*B*—H8*B*⋯O1*C* ^ii^	0.95	2.54	3.417 (19)	154
C9*B*—H9*B*⋯O2*A* ^ii^	0.95	2.54	3.274 (6)	134
C14*B*—H14*B*⋯O2*A* ^iii^	0.95	2.54	3.247 (5)	131

Symmetry codes: (i) 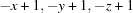; (ii) 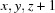; (iii) 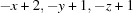.

**Fig. 1.**
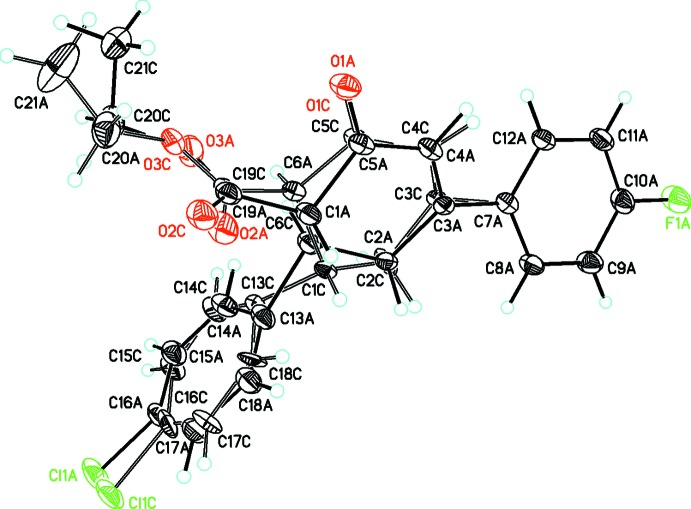
The molecular structure of molecule A in (I) showing 30% probability displacement ellipsoids. Disorder (shown as open bonds) is modeled for the 4-chlorophyenyl and oxocyclohex-3-ene rings and carboxylate and ethyl groups, with an occupancy ratio of 0.684 (5) : 0.316 (5).

**Fig. 2.**
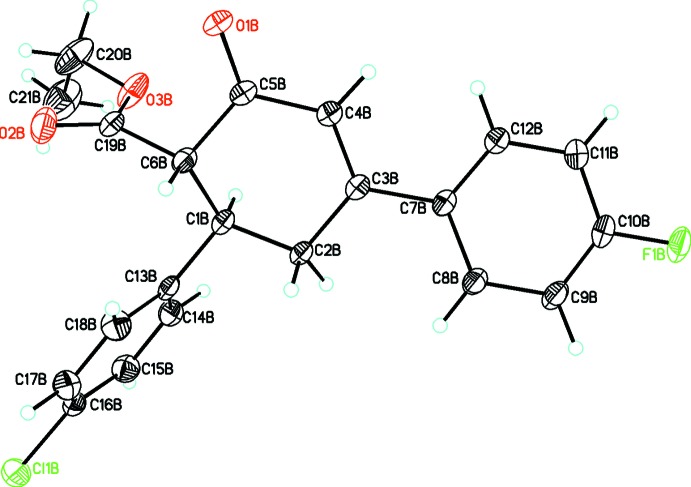
The molecular structure of molecule B in (I) showing 30% probability displacement ellipsoids.

**Fig. 3.**
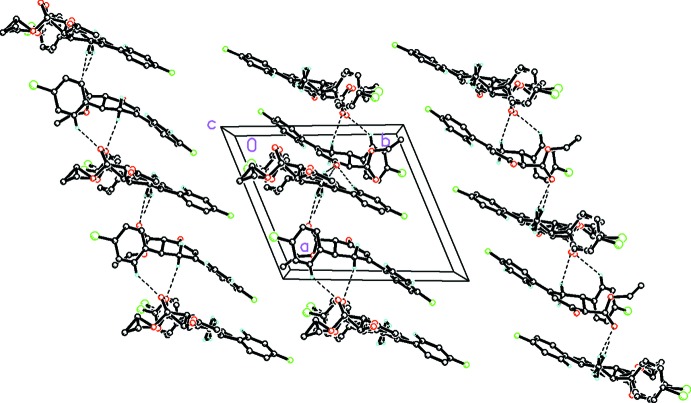
Partial packing plot for (I) viewed along the c axis. Dashed lines indicate weak C—H···O intermolecular interactions forming chains along [100]. H atoms not involved in these weak intermolecular interactions have been removed for clarity.

**Fig. 4.**
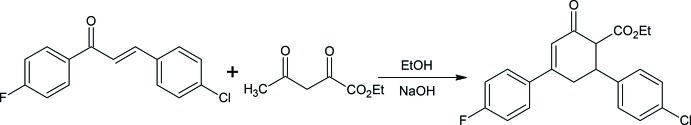
Synthesis scheme for (I).

## supplementary crystallographic information

### 1. Comment

Michael addition of ethyl acetoacetate to chalcones yield 4,6-diaryl-2-oxo-cyclohex-3-ene-1-carboxylate derivatives (Ashalatha *et al.*, 2009; Sreevidya *et al.*, 2010), which could be used as efficient synthons for building spiro compounds or as intermediates in the synthesis of isoxazoles, pyrazoles and quinazolins (Padmavathi *et al.*, 2000; Senguttuvan & Nagarajan, 2010; Butcher *et al.*, 2011). The crystal structure of some cyclohexenone derivatives, viz., methyl 4,6-bis(4-fluorophenyl)-2-oxocyclohex-3-ene-1-carboxylate (Fun *et al.*, 2010), (1RS,6SR)-ethyl 4-(4-chlorophenyl)- 6-(4-fluorophenyl)-2-oxocyclohex-3-ene-1-carboxylate toluene hemisolvate (Dutkiewicz *et al.* 2011*a*), (1RS,6SR)-ethyl 4,6-bis(4-fluorophenyl)-2-oxocyclohex-3-ene-1-carboxylate (Dutkiewicz *et al.* 2011*b*), (1RS,6SR)-ethyl 4-(2, 4-dichlorophenyl)-6-(4-fluorophenyl)-2-oxocyclohex-3-ene- 1-carboxylate (Dutkiewicz *et al.* 2011*c*), ethyl 4-(2,4- dichlorophenyl)-6-(6-methoxy-2-naphthyl)-2-oxocyclohex-3-ene- 1-carboxylate (Harrison *et al.*, 2010), ethyl 6-(4- chlorophenyl)-4-(4-fluorophenyl)-2-oxocyclohex-3-ene-1-carboxylate (Kant *et al.*, 2012) have been reported. In view of the importance of cyclohexenone derivatives, the title compound, was prepared (Fig. 4) and its crystal structure is reported.

The title compound, (I), crystallizes with two independent molecules (A & B) in the asymmetric unit (Fig. 1 & 2). Disorder is modeled for the 4-chlorophyenyl and oxocyclohex-3-ene rings and carboxylate and ethyl groups in molecule A with an occupancy ratio of 0.684 (5) : 0.316 (5) (Fig. 1). The cyclohexene ring in disordered molecule A (0.684 (5) occupancy) is in a slightly distorted envelope conformation (puckering parameters, C1A–C6A: Q, θ, and φ = 0.477 (7)Å, 57.3 (14)° and 335.4 (15)°) while in disordered molecule C (0.316 (5) occupancy) it is in a screw-boat conformation (C1C–C6C: 0.579 (17)Å, 112 (2)° and 154 (2)°. In molecule B the cyclohexene ring is in a half-chair conformation (puckering parameters, C1B–C6B: Q, θ, and φ = 0.477 (2) Å, 50.6 (3))° and 356.2 (4)°), respectively, (Cremer & Pople, 1975) (Fig. 2). Bond lengths are in normal ranges (Allen *et al.*, 1987). The dihedral angle between the mean planes of the 4-fluorophenyl and 4-chlorophenyl rings is 89.9 (7)° (molecule A) and 76.4 (7)° (molecule B), respectively. In the crystal, weak C—H···O intermolecular interactions are observed (Table 1) forming chains along [100] and which contribute to packing stability (Fig. 3).

### 2. Experimental

(2E)-3-(4-Chlorophenyl)-1-(4-fluorophenyl)prop-2-en-1-one (2.60 g, 0.01 mol) and ethyl acetoacetate (1.30 g, 0.01 mol) were refluxed for 8 hrs in 30 ml absolute alcohol in presence 10% NaOH (Fig.4). The reaction mixture was cooled to room temperature and the precipitate obtained was filtered. Single crystals were grown by slow evaporation from the solvent absolute alcohol (M.P.: 412–414 K).

### 3. Refinement

All of the H atoms were placed in their calculated positions and then refined using the riding model with Atom—H lengths of 0.95Å (CH), 0.99Å (CH_2_) 0.96Å or (CH_3_). Isotropic displacement parameters for these atoms were set to 1.2 (CH, CH_2_) or 1.5 (CH_3_) times *U*_eq_ of the parent atom. Idealised Me refined as rotating groups. Disorder was modeled for the chloro (Cl1A, Cl1C) and ethyl (C20A, C20C, C21A, C12C) groups as well as the cyclohexane ring (C1A, C1C, C2A, C2C, C3A, C3C, C4A, C4C, C5A, C5C, C6A, C6C) and carboxylate groups in molecule A with an occupancy ratio of 0.684 (5) : 0.316 (5).

### Crystal data

**Table:**

C_21_H_18_ClFO_3_	*Z* = 4
*M**_r_* = 372.80	*F*(000) = 776
Triclinic, *P*1	*D*_x_ = 1.354 Mg m^−^^3^
*a* = 11.6611 (5) Å	Cu *K*α radiation, λ = 1.54178 Å
*b* = 13.1823 (5) Å	Cell parameters from 6709 reflections
*c* = 13.2251 (5) Å	θ = 3.4–75.5°
α = 77.250 (3)°	µ = 2.09 mm^−^^1^
β = 87.320 (3)°	*T* = 123 K
γ = 67.342 (4)°	Prism, colourless
*V* = 1828.15 (14) Å^3^	0.35 × 0.23 × 0.18 mm

### Data collection

**Table:**

Agilent Xcalibur (Ruby, Gemini) diffractometer	6861 reflections with *I* > 2σ(*I*)
Detector resolution: 10.5081 pixels mm^-1^	*R*_int_ = 0.021
ω scans	θ_max_ = 75.7°, θ_min_ = 3.4°
Absorption correction: multi-scan (*CrysAlis PRO* and *CrysAlis RED*; Agilent, 2012)	*h* = −14→13
*T*_min_ = 0.508, *T*_max_ = 1.000	*k* = −16→13
13437 measured reflections	*l* = −16→12
7365 independent reflections	

### Refinement

**Table:**

Refinement on *F*^2^	Primary atom site location: structure-invariant direct methods
Least-squares matrix: full	Hydrogen site location: inferred from neighbouring sites
*R*[*F*^2^ > 2σ(*F*^2^)] = 0.055	H-atom parameters constrained
*wR*(*F*^2^) = 0.148	*w* = 1/[σ^2^(*F*_o_^2^) + (0.0631*P*)^2^ + 1.2352*P*] where *P* = (*F*_o_^2^ + 2*F*_c_^2^)/3
*S* = 1.09	(Δ/σ)_max_ < 0.001
7365 reflections	Δρ_max_ = 0.68 e Å^−^^3^
617 parameters	Δρ_min_ = −0.40 e Å^−^^3^
177 restraints	

### Special details

**Table:**

Geometry. All esds (except the esd in the dihedral angle between two l.s. planes) are estimated using the full covariance matrix. The cell esds are taken into account individually in the estimation of esds in distances, angles and torsion angles; correlations between esds in cell parameters are only used when they are defined by crystal symmetry. An approximate (isotropic) treatment of cell esds is used for estimating esds involving l.s. planes.

### Fractional atomic coordinates and isotropic or equivalent isotropic displacement parameters (Å^2^)

**Table:**

	*x*	*y*	*z*	*U*_iso_*/*U*_eq_	Occ. (<1)
F1A	0.40903 (18)	0.18316 (15)	0.39705 (12)	0.0598 (4)	
C7A	0.55724 (18)	0.41724 (17)	0.26781 (15)	0.0298 (4)	
C8A	0.5667 (2)	0.37490 (19)	0.37517 (16)	0.0347 (4)	
H8A	0.6072	0.4008	0.4184	0.042*	
C9A	0.5176 (2)	0.2955 (2)	0.41917 (17)	0.0418 (5)	
H9A	0.5254	0.2658	0.4919	0.050*	
C10A	0.4575 (2)	0.2607 (2)	0.35531 (19)	0.0409 (5)	
C11A	0.4429 (2)	0.3018 (2)	0.24975 (18)	0.0381 (5)	
H11A	0.3993	0.2773	0.2078	0.046*	
C12A	0.4937 (2)	0.38005 (18)	0.20622 (16)	0.0336 (4)	
H12A	0.4853	0.4090	0.1333	0.040*	
C13A	0.6987 (4)	0.7320 (5)	0.2915 (6)	0.0352 (12)	0.684 (5)
C14A	0.5941 (4)	0.8237 (6)	0.3060 (7)	0.0375 (10)	0.684 (5)
H14A	0.5150	0.8352	0.2786	0.045*	0.684 (5)
C15A	0.6050 (5)	0.8986 (7)	0.3605 (8)	0.0410 (14)	0.684 (5)
H15A	0.5335	0.9613	0.3704	0.049*	0.684 (5)
C16A	0.7207 (5)	0.8818 (6)	0.4005 (8)	0.0376 (13)	0.684 (5)
C17A	0.8253 (4)	0.7901 (6)	0.3860 (6)	0.0403 (10)	0.684 (5)
H17A	0.9044	0.7786	0.4134	0.048*	0.684 (5)
C18A	0.8144 (4)	0.7152 (4)	0.3315 (5)	0.0390 (11)	0.684 (5)
H18A	0.8859	0.6525	0.3216	0.047*	0.684 (5)
C13C	0.6632 (8)	0.7550 (10)	0.2832 (12)	0.0214 (16)	0.316 (5)
C14C	0.5752 (8)	0.8455 (13)	0.3173 (15)	0.0339 (19)	0.316 (5)
H14C	0.4899	0.8706	0.2971	0.041*	0.316 (5)
C15C	0.6120 (11)	0.8994 (15)	0.3810 (17)	0.038 (2)	0.316 (5)
H15C	0.5519	0.9613	0.4044	0.046*	0.316 (5)
C16C	0.7368 (12)	0.8628 (15)	0.4106 (17)	0.041 (3)	0.316 (5)
C17C	0.8248 (9)	0.7722 (14)	0.3765 (16)	0.045 (3)	0.316 (5)
H17C	0.9101	0.7472	0.3967	0.054*	0.316 (5)
C18C	0.7880 (8)	0.7183 (11)	0.3127 (12)	0.036 (2)	0.316 (5)
H18C	0.8481	0.6565	0.2894	0.043*	0.316 (5)
O1A	0.7088 (8)	0.6061 (9)	−0.0287 (5)	0.039 (2)	0.684 (5)
O2A	0.8622 (4)	0.7464 (5)	0.0453 (4)	0.0443 (9)	0.684 (5)
O3A	0.6551 (4)	0.8353 (4)	0.0286 (5)	0.0460 (9)	0.684 (5)
C1A	0.6422 (4)	0.6813 (3)	0.2171 (2)	0.0323 (7)	0.684 (5)
H1A	0.5598	0.7378	0.1856	0.039*	0.684 (5)
C2A	0.6284 (5)	0.5752 (5)	0.2825 (5)	0.0254 (11)	0.684 (5)
H2AA	0.7008	0.5344	0.3323	0.030*	0.684 (5)
H2AB	0.5529	0.5985	0.3233	0.030*	0.684 (5)
C3A	0.6191 (10)	0.4952 (7)	0.2200 (6)	0.0232 (17)	0.684 (5)
C4A	0.6432 (19)	0.5094 (11)	0.1178 (8)	0.033 (3)	0.684 (5)
H4A	0.6247	0.4650	0.0786	0.040*	0.684 (5)
C5A	0.6963 (16)	0.5895 (10)	0.0651 (6)	0.031 (2)	0.684 (5)
C6A	0.7355 (4)	0.6512 (3)	0.1330 (2)	0.0323 (7)	0.684 (5)
H6A	0.8164	0.5971	0.1691	0.039*	0.684 (5)
C19A	0.7606 (5)	0.7482 (7)	0.0639 (9)	0.0326 (13)	0.684 (5)
C20A	0.6677 (5)	0.9329 (3)	−0.0439 (3)	0.0526 (10)	0.684 (5)
H20A	0.7443	0.9414	−0.0253	0.063*	0.684 (5)
H20B	0.5957	1.0026	−0.0390	0.063*	0.684 (5)
C21A	0.6735 (5)	0.9152 (4)	−0.1535 (3)	0.0620 (12)	0.684 (5)
H21A	0.6818	0.9800	−0.2016	0.093*	0.684 (5)
H21B	0.5972	0.9076	−0.1718	0.093*	0.684 (5)
H21C	0.7454	0.8466	−0.1582	0.093*	0.684 (5)
Cl1A	0.7356 (3)	0.9740 (2)	0.4685 (2)	0.0659 (7)	0.684 (5)
O1C	0.707 (2)	0.609 (2)	−0.0248 (11)	0.046 (5)	0.316 (5)
O2C	0.8488 (8)	0.7264 (10)	0.0760 (8)	0.0437 (19)	0.316 (5)
O3C	0.6643 (10)	0.8545 (9)	0.0056 (10)	0.046 (2)	0.316 (5)
C1C	0.7108 (6)	0.6376 (5)	0.2355 (4)	0.0224 (11)	0.316 (5)
H1C	0.8020	0.5908	0.2446	0.027*	0.316 (5)
C2C	0.6260 (19)	0.5767 (16)	0.2845 (12)	0.045 (4)	0.316 (5)
H2CA	0.6674	0.5234	0.3496	0.054*	0.316 (5)
H2CB	0.5479	0.6332	0.3029	0.054*	0.316 (5)
C3C	0.593 (2)	0.5124 (13)	0.2163 (12)	0.017 (3)	0.316 (5)
C4C	0.640 (4)	0.511 (2)	0.1206 (16)	0.027 (6)	0.316 (5)
H4C	0.6521	0.4486	0.0903	0.033*	0.316 (5)
C5C	0.672 (3)	0.604 (2)	0.0641 (12)	0.026 (4)	0.316 (5)
C6C	0.6624 (6)	0.6939 (5)	0.1230 (4)	0.0215 (12)	0.316 (5)
H6C	0.5735	0.7465	0.1224	0.026*	0.316 (5)
C19C	0.7385 (9)	0.7589 (13)	0.0677 (19)	0.030 (2)	0.316 (5)
C20C	0.7380 (9)	0.9112 (7)	−0.0596 (7)	0.052 (2)	0.316 (5)
H20C	0.7982	0.8604	−0.0996	0.062*	0.316 (5)
H20D	0.7834	0.9380	−0.0171	0.062*	0.316 (5)
C21C	0.6394 (13)	1.0084 (12)	−0.1302 (11)	0.106 (6)	0.316 (5)
H21D	0.6779	1.0554	−0.1747	0.159*	0.316 (5)
H21E	0.5775	1.0537	−0.0883	0.159*	0.316 (5)
H21F	0.5987	0.9794	−0.1734	0.159*	0.316 (5)
Cl1C	0.7756 (6)	0.9386 (5)	0.4862 (5)	0.0558 (11)	0.316 (5)
Cl1B	0.71926 (7)	0.05408 (6)	1.16767 (5)	0.05522 (19)	
F1B	1.15955 (14)	0.76617 (13)	0.77809 (13)	0.0508 (4)	
O1B	0.75978 (18)	0.44532 (17)	0.51477 (12)	0.0490 (4)	
O2B	0.66040 (19)	0.2488 (2)	0.66133 (17)	0.0602 (5)	
O3B	0.86198 (17)	0.20485 (17)	0.63467 (17)	0.0551 (5)	
C1B	0.8480 (2)	0.33555 (18)	0.78861 (16)	0.0326 (4)	
H1B	0.9315	0.2867	0.7687	0.039*	
C2B	0.86005 (19)	0.44019 (17)	0.81283 (15)	0.0298 (4)	
H2BA	0.7821	0.4843	0.8426	0.036*	
H2BB	0.9283	0.4155	0.8657	0.036*	
C3B	0.88570 (19)	0.51490 (17)	0.71829 (16)	0.0298 (4)	
C4B	0.8495 (2)	0.51634 (19)	0.62257 (16)	0.0342 (4)	
H4B	0.8645	0.5668	0.5652	0.041*	
C5B	0.7886 (2)	0.44449 (19)	0.60262 (16)	0.0348 (4)	
C6B	0.7566 (2)	0.37018 (18)	0.69533 (16)	0.0328 (4)	
H6B	0.6718	0.4144	0.7166	0.039*	
C7B	0.95342 (19)	0.58550 (17)	0.73334 (16)	0.0308 (4)	
C8B	0.9539 (2)	0.61594 (19)	0.82868 (17)	0.0354 (4)	
H8B	0.9068	0.5940	0.8835	0.042*	
C9B	1.0224 (2)	0.6777 (2)	0.84368 (19)	0.0400 (5)	
H9B	1.0226	0.6983	0.9081	0.048*	
C10B	1.0901 (2)	0.70842 (19)	0.76335 (19)	0.0387 (4)	
C11B	1.0919 (2)	0.68128 (19)	0.66812 (18)	0.0379 (4)	
H11B	1.1387	0.7043	0.6137	0.046*	
C12B	1.0233 (2)	0.61943 (18)	0.65422 (17)	0.0345 (4)	
H12B	1.0239	0.5996	0.5893	0.041*	
C13B	0.8118 (2)	0.26734 (18)	0.88381 (16)	0.0321 (4)	
C14B	0.9047 (2)	0.1782 (2)	0.94658 (17)	0.0387 (4)	
H14B	0.9890	0.1612	0.9292	0.046*	
C15B	0.8772 (2)	0.1132 (2)	1.03424 (19)	0.0433 (5)	
H15B	0.9420	0.0525	1.0767	0.052*	
C16B	0.7550 (2)	0.13739 (19)	1.05912 (17)	0.0378 (4)	
C17B	0.6602 (2)	0.2265 (2)	0.99981 (19)	0.0440 (5)	
H17B	0.5761	0.2438	1.0182	0.053*	
C18B	0.6900 (2)	0.2909 (2)	0.91231 (19)	0.0427 (5)	
H18B	0.6250	0.3527	0.8710	0.051*	
C19B	0.7517 (2)	0.2683 (2)	0.66292 (17)	0.0375 (5)	
C20B	0.8715 (4)	0.1043 (2)	0.6009 (3)	0.0696 (9)	
H20E	0.7871	0.1070	0.5889	0.083*	
H20F	0.9145	0.1016	0.5346	0.083*	
C21B	0.9387 (5)	0.0056 (3)	0.6773 (4)	0.0896 (13)	
H21G	0.9443	−0.0613	0.6534	0.134*	
H21H	0.8954	0.0079	0.7426	0.134*	
H21I	1.0226	0.0026	0.6884	0.134*	

### Atomic displacement parameters (Å^2^)

**Table:**

	*U*^11^	*U*^22^	*U*^33^	*U*^12^	*U*^13^	*U*^23^
F1A	0.0891 (12)	0.0722 (11)	0.0449 (8)	−0.0633 (10)	0.0047 (8)	−0.0074 (7)
C7A	0.0313 (10)	0.0315 (9)	0.0305 (10)	−0.0139 (8)	0.0003 (7)	−0.0113 (8)
C8A	0.0403 (11)	0.0430 (11)	0.0294 (10)	−0.0232 (9)	−0.0006 (8)	−0.0113 (8)
C9A	0.0529 (13)	0.0518 (13)	0.0297 (10)	−0.0312 (11)	0.0008 (9)	−0.0068 (9)
C10A	0.0513 (13)	0.0442 (12)	0.0399 (12)	−0.0322 (11)	0.0054 (10)	−0.0095 (9)
C11A	0.0458 (12)	0.0422 (11)	0.0369 (11)	−0.0252 (10)	−0.0013 (9)	−0.0138 (9)
C12A	0.0413 (11)	0.0364 (10)	0.0293 (10)	−0.0193 (9)	−0.0014 (8)	−0.0105 (8)
C13A	0.050 (3)	0.041 (3)	0.028 (2)	−0.029 (2)	0.008 (3)	−0.015 (3)
C14A	0.043 (2)	0.052 (3)	0.032 (3)	−0.0284 (19)	0.007 (2)	−0.020 (2)
C15A	0.054 (2)	0.044 (2)	0.034 (4)	−0.0249 (17)	0.0059 (17)	−0.0169 (19)
C16A	0.065 (3)	0.042 (3)	0.023 (2)	−0.0368 (17)	0.0041 (19)	−0.012 (2)
C17A	0.053 (2)	0.048 (2)	0.034 (3)	−0.0334 (17)	−0.0010 (19)	−0.0107 (18)
C18A	0.046 (2)	0.042 (2)	0.038 (3)	−0.0242 (18)	0.003 (2)	−0.015 (2)
C13C	0.022 (4)	0.022 (4)	0.022 (3)	−0.010 (3)	−0.003 (3)	−0.006 (3)
C14C	0.045 (3)	0.040 (5)	0.029 (5)	−0.021 (3)	0.016 (4)	−0.027 (4)
C15C	0.060 (4)	0.048 (5)	0.025 (7)	−0.033 (4)	0.015 (3)	−0.024 (4)
C16C	0.064 (4)	0.044 (6)	0.040 (8)	−0.044 (4)	0.011 (5)	−0.017 (6)
C17C	0.049 (4)	0.056 (7)	0.049 (6)	−0.033 (4)	−0.007 (4)	−0.025 (5)
C18C	0.032 (3)	0.048 (4)	0.034 (5)	−0.016 (3)	−0.016 (4)	−0.016 (3)
O1A	0.046 (4)	0.054 (5)	0.028 (2)	−0.026 (3)	0.004 (2)	−0.016 (3)
O2A	0.0440 (16)	0.057 (2)	0.043 (3)	−0.0312 (13)	0.0039 (15)	−0.0099 (17)
O3A	0.0471 (15)	0.038 (2)	0.056 (2)	−0.0202 (15)	0.0004 (15)	−0.0083 (13)
C1A	0.0406 (19)	0.0360 (16)	0.0286 (14)	−0.0215 (15)	0.0030 (12)	−0.0116 (11)
C2A	0.024 (2)	0.028 (2)	0.0242 (19)	−0.0085 (19)	−0.0038 (18)	−0.0080 (15)
C3A	0.013 (4)	0.021 (2)	0.0300 (18)	0.000 (3)	−0.0071 (18)	−0.0074 (15)
C4A	0.041 (9)	0.035 (5)	0.031 (3)	−0.018 (5)	−0.001 (5)	−0.015 (4)
C5A	0.033 (7)	0.036 (3)	0.0280 (17)	−0.014 (4)	−0.0021 (17)	−0.0123 (15)
C6A	0.0381 (19)	0.0385 (15)	0.0288 (13)	−0.0208 (15)	0.0016 (11)	−0.0129 (10)
C19A	0.041 (2)	0.039 (2)	0.026 (2)	−0.0209 (18)	0.001 (2)	−0.0118 (19)
C20A	0.059 (3)	0.0339 (17)	0.064 (2)	−0.0195 (19)	−0.004 (2)	−0.0062 (15)
C21A	0.079 (3)	0.050 (2)	0.0546 (18)	−0.030 (2)	−0.020 (2)	0.0083 (17)
Cl1A	0.112 (2)	0.0687 (14)	0.0535 (11)	−0.0643 (14)	0.0104 (11)	−0.0329 (11)
O1C	0.090 (13)	0.046 (10)	0.019 (5)	−0.043 (9)	0.007 (6)	−0.012 (5)
O2C	0.038 (3)	0.062 (5)	0.046 (6)	−0.035 (3)	0.015 (3)	−0.013 (4)
O3C	0.063 (4)	0.046 (4)	0.044 (5)	−0.039 (3)	−0.009 (3)	−0.004 (2)
C1C	0.016 (3)	0.026 (3)	0.026 (2)	−0.006 (2)	−0.004 (2)	−0.0111 (18)
C2C	0.086 (12)	0.051 (8)	0.027 (4)	−0.053 (9)	0.015 (7)	−0.019 (5)
C3C	0.003 (7)	0.018 (5)	0.024 (4)	0.003 (5)	−0.005 (3)	−0.007 (3)
C4C	0.028 (15)	0.038 (9)	0.024 (7)	−0.019 (11)	0.000 (9)	−0.012 (7)
C5C	0.026 (11)	0.033 (6)	0.024 (3)	−0.013 (8)	0.002 (4)	−0.012 (3)
C6C	0.013 (3)	0.028 (3)	0.024 (2)	−0.006 (2)	0.0021 (18)	−0.0106 (17)
C19C	0.036 (4)	0.040 (4)	0.027 (4)	−0.023 (3)	0.012 (4)	−0.020 (3)
C20C	0.066 (6)	0.045 (4)	0.049 (4)	−0.027 (4)	0.016 (4)	−0.010 (3)
C21C	0.105 (9)	0.090 (10)	0.087 (9)	−0.028 (7)	0.000 (7)	0.040 (7)
Cl1C	0.081 (3)	0.062 (3)	0.052 (2)	−0.047 (2)	0.0040 (16)	−0.031 (2)
Cl1B	0.0809 (5)	0.0581 (4)	0.0389 (3)	−0.0437 (4)	0.0089 (3)	−0.0049 (3)
F1B	0.0528 (8)	0.0499 (8)	0.0685 (10)	−0.0334 (7)	0.0044 (7)	−0.0258 (7)
O1B	0.0697 (12)	0.0654 (11)	0.0292 (7)	−0.0419 (10)	−0.0028 (7)	−0.0146 (7)
O2B	0.0589 (11)	0.0798 (14)	0.0713 (13)	−0.0472 (11)	0.0159 (10)	−0.0402 (11)
O3B	0.0458 (10)	0.0546 (11)	0.0733 (13)	−0.0136 (8)	−0.0072 (9)	−0.0396 (10)
C1B	0.0375 (10)	0.0359 (10)	0.0316 (9)	−0.0186 (8)	−0.0025 (7)	−0.0126 (7)
C2B	0.0351 (10)	0.0331 (9)	0.0271 (8)	−0.0168 (8)	−0.0020 (7)	−0.0106 (7)
C3B	0.0314 (9)	0.0306 (9)	0.0305 (9)	−0.0126 (7)	−0.0007 (7)	−0.0111 (7)
C4B	0.0421 (11)	0.0386 (11)	0.0277 (9)	−0.0209 (9)	−0.0005 (8)	−0.0084 (8)
C5B	0.0408 (11)	0.0416 (11)	0.0284 (8)	−0.0196 (9)	−0.0008 (8)	−0.0131 (8)
C6B	0.0351 (10)	0.0393 (11)	0.0311 (8)	−0.0184 (8)	0.0003 (7)	−0.0144 (7)
C7B	0.0335 (10)	0.0296 (9)	0.0326 (9)	−0.0137 (8)	−0.0016 (7)	−0.0103 (7)
C8B	0.0388 (11)	0.0392 (11)	0.0364 (10)	−0.0196 (9)	0.0033 (8)	−0.0168 (9)
C9B	0.0422 (12)	0.0442 (12)	0.0451 (11)	−0.0214 (9)	0.0031 (9)	−0.0240 (10)
C10B	0.0378 (11)	0.0326 (10)	0.0527 (12)	−0.0176 (9)	−0.0018 (9)	−0.0152 (9)
C11B	0.0403 (11)	0.0350 (11)	0.0432 (10)	−0.0199 (9)	0.0019 (9)	−0.0080 (9)
C12B	0.0394 (11)	0.0358 (10)	0.0331 (9)	−0.0183 (8)	−0.0003 (8)	−0.0096 (8)
C13B	0.0397 (10)	0.0356 (9)	0.0309 (9)	−0.0217 (8)	−0.0032 (7)	−0.0127 (7)
C14B	0.0364 (10)	0.0474 (12)	0.0361 (10)	−0.0200 (9)	−0.0028 (8)	−0.0085 (8)
C15B	0.0473 (11)	0.0434 (12)	0.0374 (11)	−0.0179 (10)	−0.0046 (9)	−0.0032 (8)
C16B	0.0545 (11)	0.0403 (11)	0.0304 (10)	−0.0301 (9)	0.0026 (8)	−0.0097 (7)
C17B	0.0417 (11)	0.0523 (13)	0.0427 (11)	−0.0228 (9)	0.0045 (9)	−0.0120 (9)
C18B	0.0392 (10)	0.0426 (12)	0.0430 (11)	−0.0145 (9)	−0.0019 (9)	−0.0044 (9)
C19B	0.0482 (12)	0.0447 (12)	0.0317 (10)	−0.0264 (10)	0.0002 (9)	−0.0161 (9)
C20B	0.085 (2)	0.0450 (15)	0.077 (2)	−0.0085 (15)	−0.0216 (17)	−0.0350 (15)
C21B	0.118 (3)	0.0538 (19)	0.094 (3)	−0.028 (2)	−0.028 (2)	−0.0130 (18)

### Geometric parameters (Å, º)

**Table:**

F1A—C10A	1.355 (3)	C1C—C2C	1.537 (6)
C7A—C8A	1.399 (3)	C1C—C6C	1.533 (5)
C7A—C12A	1.402 (3)	C2C—H2CA	0.9900
C7A—C3A	1.490 (4)	C2C—H2CB	0.9900
C7A—C3C	1.491 (5)	C2C—C3C	1.513 (5)
C8A—H8A	0.9500	C3C—C4C	1.355 (6)
C8A—C9A	1.387 (3)	C4C—H4C	0.9500
C9A—H9A	0.9500	C4C—C5C	1.456 (6)
C9A—C10A	1.374 (3)	C5C—C6C	1.526 (6)
C10A—C11A	1.374 (3)	C6C—H6C	1.0000
C11A—H11A	0.9500	C6C—C19C	1.518 (5)
C11A—C12A	1.387 (3)	C20C—H20C	0.9900
C12A—H12A	0.9500	C20C—H20D	0.9900
C13A—C14A	1.3900	C20C—C21C	1.508 (7)
C13A—C18A	1.3900	C21C—H21D	0.9800
C13A—C1A	1.593 (4)	C21C—H21E	0.9800
C14A—H14A	0.9500	C21C—H21F	0.9800
C14A—C15A	1.3900	Cl1B—C16B	1.744 (2)
C15A—H15A	0.9500	F1B—C10B	1.352 (2)
C15A—C16A	1.3900	O1B—C5B	1.221 (3)
C16A—C17A	1.3900	O2B—C19B	1.189 (3)
C16A—Cl1A	1.724 (3)	O3B—C19B	1.326 (3)
C17A—H17A	0.9500	O3B—C20B	1.453 (3)
C17A—C18A	1.3900	C1B—H1B	1.0000
C18A—H18A	0.9500	C1B—C2B	1.541 (3)
C13C—C14C	1.3900	C1B—C6B	1.538 (3)
C13C—C18C	1.3900	C1B—C13B	1.522 (3)
C13C—C1C	1.684 (9)	C2B—H2BA	0.9900
C14C—H14C	0.9500	C2B—H2BB	0.9900
C14C—C15C	1.3900	C2B—C3B	1.507 (3)
C15C—H15C	0.9500	C3B—C4B	1.347 (3)
C15C—C16C	1.3900	C3B—C7B	1.481 (3)
C16C—C17C	1.3900	C4B—H4B	0.9500
C16C—Cl1C	1.742 (7)	C4B—C5B	1.456 (3)
C17C—H17C	0.9500	C5B—C6B	1.520 (3)
C17C—C18C	1.3900	C6B—H6B	1.0000
C18C—H18C	0.9500	C6B—C19B	1.519 (3)
O1A—C5A	1.224 (5)	C7B—C8B	1.406 (3)
O2A—C19A	1.190 (5)	C7B—C12B	1.398 (3)
O3A—C19A	1.333 (5)	C8B—H8B	0.9500
O3A—C20A	1.478 (5)	C8B—C9B	1.388 (3)
C1A—H1A	1.0000	C9B—H9B	0.9500
C1A—C2A	1.536 (4)	C9B—C10B	1.376 (3)
C1A—C6A	1.525 (4)	C10B—C11B	1.380 (3)
C2A—H2AA	0.9900	C11B—H11B	0.9500
C2A—H2AB	0.9900	C11B—C12B	1.385 (3)
C2A—C3A	1.513 (4)	C12B—H12B	0.9500
C3A—C4A	1.354 (5)	C13B—C14B	1.384 (3)
C4A—H4A	0.9500	C13B—C18B	1.385 (3)
C4A—C5A	1.455 (5)	C14B—H14B	0.9500
C5A—C6A	1.525 (6)	C14B—C15B	1.385 (3)
C6A—H6A	1.0000	C15B—H15B	0.9500
C6A—C19A	1.519 (4)	C15B—C16B	1.376 (4)
C20A—H20A	0.9900	C16B—C17B	1.375 (4)
C20A—H20B	0.9900	C17B—H17B	0.9500
C20A—C21A	1.513 (6)	C17B—C18B	1.391 (3)
C21A—H21A	0.9800	C18B—H18B	0.9500
C21A—H21B	0.9800	C20B—H20E	0.9900
C21A—H21C	0.9800	C20B—H20F	0.9900
O1C—C5C	1.224 (6)	C20B—C21B	1.427 (5)
O2C—C19C	1.191 (6)	C21B—H21G	0.9800
O3C—C19C	1.334 (6)	C21B—H21H	0.9800
O3C—C20C	1.477 (6)	C21B—H21I	0.9800
C1C—H1C	1.0000		
			
C8A—C7A—C12A	118.40 (19)	C3C—C2C—H2CB	108.5
C8A—C7A—C3A	120.5 (4)	C7A—C3C—C2C	118.0 (9)
C8A—C7A—C3C	123.7 (7)	C4C—C3C—C7A	117.5 (11)
C12A—C7A—C3A	121.0 (4)	C4C—C3C—C2C	119.9 (10)
C12A—C7A—C3C	117.2 (7)	C3C—C4C—H4C	119.9
C7A—C8A—H8A	119.6	C3C—C4C—C5C	120.2 (13)
C9A—C8A—C7A	120.72 (19)	C5C—C4C—H4C	119.9
C9A—C8A—H8A	119.6	O1C—C5C—C4C	122.1 (13)
C8A—C9A—H9A	120.7	O1C—C5C—C6C	121.6 (13)
C10A—C9A—C8A	118.6 (2)	C4C—C5C—C6C	116.3 (11)
C10A—C9A—H9A	120.7	C1C—C6C—H6C	109.3
F1A—C10A—C9A	119.4 (2)	C5C—C6C—C1C	109.5 (11)
F1A—C10A—C11A	117.7 (2)	C5C—C6C—H6C	109.3
C9A—C10A—C11A	122.9 (2)	C19C—C6C—C1C	111.4 (9)
C10A—C11A—H11A	121.0	C19C—C6C—C5C	107.9 (13)
C10A—C11A—C12A	118.1 (2)	C19C—C6C—H6C	109.3
C12A—C11A—H11A	121.0	O2C—C19C—O3C	125.5 (8)
C7A—C12A—H12A	119.4	O2C—C19C—C6C	124.4 (8)
C11A—C12A—C7A	121.2 (2)	O3C—C19C—C6C	110.1 (7)
C11A—C12A—H12A	119.4	O3C—C20C—H20C	111.3
C14A—C13A—C18A	120.0	O3C—C20C—H20D	111.3
C14A—C13A—C1A	101.1 (3)	O3C—C20C—C21C	102.4 (8)
C18A—C13A—C1A	138.6 (3)	H20C—C20C—H20D	109.2
C13A—C14A—H14A	120.0	C21C—C20C—H20C	111.3
C15A—C14A—C13A	120.0	C21C—C20C—H20D	111.3
C15A—C14A—H14A	120.0	C20C—C21C—H21D	109.5
C14A—C15A—H15A	120.0	C20C—C21C—H21E	109.5
C14A—C15A—C16A	120.0	C20C—C21C—H21F	109.5
C16A—C15A—H15A	120.0	H21D—C21C—H21E	109.5
C15A—C16A—C17A	120.0	H21D—C21C—H21F	109.5
C15A—C16A—Cl1A	120.5 (2)	H21E—C21C—H21F	109.5
C17A—C16A—Cl1A	119.5 (2)	C19B—O3B—C20B	117.5 (2)
C16A—C17A—H17A	120.0	C2B—C1B—H1B	107.6
C16A—C17A—C18A	120.0	C6B—C1B—H1B	107.6
C18A—C17A—H17A	120.0	C6B—C1B—C2B	110.35 (17)
C13A—C18A—H18A	120.0	C13B—C1B—H1B	107.6
C17A—C18A—C13A	120.0	C13B—C1B—C2B	111.21 (16)
C17A—C18A—H18A	120.0	C13B—C1B—C6B	112.24 (17)
C14C—C13C—C18C	120.0	C1B—C2B—H2BA	109.0
C14C—C13C—C1C	154.5 (6)	C1B—C2B—H2BB	109.0
C18C—C13C—C1C	84.1 (5)	H2BA—C2B—H2BB	107.8
C13C—C14C—H14C	120.0	C3B—C2B—C1B	112.74 (16)
C13C—C14C—C15C	120.0	C3B—C2B—H2BA	109.0
C15C—C14C—H14C	120.0	C3B—C2B—H2BB	109.0
C14C—C15C—H15C	120.0	C4B—C3B—C2B	120.95 (18)
C16C—C15C—C14C	120.0	C4B—C3B—C7B	120.86 (19)
C16C—C15C—H15C	120.0	C7B—C3B—C2B	118.18 (17)
C15C—C16C—C17C	120.0	C3B—C4B—H4B	118.4
C15C—C16C—Cl1C	117.0 (6)	C3B—C4B—C5B	123.3 (2)
C17C—C16C—Cl1C	123.0 (6)	C5B—C4B—H4B	118.4
C16C—C17C—H17C	120.0	O1B—C5B—C4B	121.9 (2)
C16C—C17C—C18C	120.0	O1B—C5B—C6B	120.09 (19)
C18C—C17C—H17C	120.0	C4B—C5B—C6B	117.97 (17)
C13C—C18C—H18C	120.0	C1B—C6B—H6B	107.9
C17C—C18C—C13C	120.0	C5B—C6B—C1B	112.02 (17)
C17C—C18C—H18C	120.0	C5B—C6B—H6B	107.9
C19A—O3A—C20A	116.4 (4)	C19B—C6B—C1B	111.75 (18)
C13A—C1A—H1A	110.6	C19B—C6B—C5B	109.21 (17)
C2A—C1A—C13A	107.7 (4)	C19B—C6B—H6B	107.9
C2A—C1A—H1A	110.6	C8B—C7B—C3B	121.13 (19)
C6A—C1A—C13A	106.7 (3)	C12B—C7B—C3B	120.62 (18)
C6A—C1A—H1A	110.6	C12B—C7B—C8B	118.19 (19)
C6A—C1A—C2A	110.6 (4)	C7B—C8B—H8B	119.6
C1A—C2A—H2AA	108.7	C9B—C8B—C7B	120.7 (2)
C1A—C2A—H2AB	108.7	C9B—C8B—H8B	119.6
H2AA—C2A—H2AB	107.6	C8B—C9B—H9B	120.7
C3A—C2A—C1A	114.3 (3)	C10B—C9B—C8B	118.7 (2)
C3A—C2A—H2AA	108.7	C10B—C9B—H9B	120.7
C3A—C2A—H2AB	108.7	F1B—C10B—C9B	119.1 (2)
C7A—C3A—C2A	117.5 (5)	F1B—C10B—C11B	118.2 (2)
C4A—C3A—C7A	120.6 (6)	C9B—C10B—C11B	122.8 (2)
C4A—C3A—C2A	120.6 (4)	C10B—C11B—H11B	121.0
C3A—C4A—H4A	118.4	C10B—C11B—C12B	118.0 (2)
C3A—C4A—C5A	123.3 (7)	C12B—C11B—H11B	121.0
C5A—C4A—H4A	118.4	C7B—C12B—H12B	119.2
O1A—C5A—C4A	121.7 (7)	C11B—C12B—C7B	121.6 (2)
O1A—C5A—C6A	121.8 (7)	C11B—C12B—H12B	119.2
C4A—C5A—C6A	116.5 (6)	C14B—C13B—C1B	118.9 (2)
C1A—C6A—H6A	106.9	C14B—C13B—C18B	117.8 (2)
C5A—C6A—C1A	110.4 (5)	C18B—C13B—C1B	123.3 (2)
C5A—C6A—H6A	106.9	C13B—C14B—H14B	119.3
C19A—C6A—C1A	116.2 (5)	C13B—C14B—C15B	121.4 (2)
C19A—C6A—C5A	109.0 (6)	C15B—C14B—H14B	119.3
C19A—C6A—H6A	106.9	C14B—C15B—H15B	120.4
O2A—C19A—O3A	124.9 (4)	C16B—C15B—C14B	119.3 (2)
O2A—C19A—C6A	123.7 (4)	C16B—C15B—H15B	120.4
O3A—C19A—C6A	111.4 (4)	C15B—C16B—Cl1B	119.63 (19)
O3A—C20A—H20A	109.8	C17B—C16B—Cl1B	119.30 (19)
O3A—C20A—H20B	109.8	C17B—C16B—C15B	121.1 (2)
O3A—C20A—C21A	109.5 (4)	C16B—C17B—H17B	120.7
H20A—C20A—H20B	108.2	C16B—C17B—C18B	118.7 (2)
C21A—C20A—H20A	109.8	C18B—C17B—H17B	120.7
C21A—C20A—H20B	109.8	C13B—C18B—C17B	121.7 (2)
C20A—C21A—H21A	109.5	C13B—C18B—H18B	119.1
C20A—C21A—H21B	109.5	C17B—C18B—H18B	119.1
C20A—C21A—H21C	109.5	O2B—C19B—O3B	124.3 (2)
H21A—C21A—H21B	109.5	O2B—C19B—C6B	124.5 (2)
H21A—C21A—H21C	109.5	O3B—C19B—C6B	111.16 (19)
H21B—C21A—H21C	109.5	O3B—C20B—H20E	109.6
C19C—O3C—C20C	110.8 (8)	O3B—C20B—H20F	109.6
C13C—C1C—H1C	115.3	H20E—C20B—H20F	108.1
C2C—C1C—C13C	104.3 (10)	C21B—C20B—O3B	110.5 (3)
C2C—C1C—H1C	115.3	C21B—C20B—H20E	109.6
C6C—C1C—C13C	97.9 (6)	C21B—C20B—H20F	109.6
C6C—C1C—H1C	115.3	C20B—C21B—H21G	109.5
C6C—C1C—C2C	107.0 (8)	C20B—C21B—H21H	109.5
C1C—C2C—H2CA	108.5	C20B—C21B—H21I	109.5
C1C—C2C—H2CB	108.5	H21G—C21B—H21H	109.5
H2CA—C2C—H2CB	107.5	H21G—C21B—H21I	109.5
C3C—C2C—C1C	114.9 (8)	H21H—C21B—H21I	109.5
C3C—C2C—H2CA	108.5		
			
F1A—C10A—C11A—C12A	−179.1 (2)	C1C—C13C—C18C—C17C	−171.2 (11)
C7A—C8A—C9A—C10A	−1.1 (4)	C1C—C2C—C3C—C7A	156.1 (14)
C7A—C3A—C4A—C5A	176.1 (14)	C1C—C2C—C3C—C4C	1 (3)
C7A—C3C—C4C—C5C	177 (3)	C1C—C6C—C19C—O2C	41 (3)
C8A—C7A—C12A—C11A	−1.0 (3)	C1C—C6C—C19C—O3C	−141.6 (17)
C8A—C7A—C3A—C2A	−33.0 (10)	C2C—C1C—C6C—C5C	−61.9 (15)
C8A—C7A—C3A—C4A	159.8 (12)	C2C—C1C—C6C—C19C	178.8 (13)
C8A—C7A—C3C—C2C	−8 (2)	C2C—C3C—C4C—C5C	−28 (5)
C8A—C7A—C3C—C4C	147 (2)	C3C—C7A—C8A—C9A	172.2 (10)
C8A—C9A—C10A—F1A	180.0 (2)	C3C—C7A—C12A—C11A	−171.9 (9)
C8A—C9A—C10A—C11A	−0.6 (4)	C3C—C7A—C3A—C2A	76 (5)
C9A—C10A—C11A—C12A	1.4 (4)	C3C—C7A—C3A—C4A	−91 (5)
C10A—C11A—C12A—C7A	−0.6 (3)	C3C—C4C—C5C—O1C	−176 (3)
C12A—C7A—C8A—C9A	1.9 (3)	C3C—C4C—C5C—C6C	6 (5)
C12A—C7A—C3A—C2A	150.2 (6)	C4C—C5C—C6C—C1C	40 (3)
C12A—C7A—C3A—C4A	−17.0 (14)	C4C—C5C—C6C—C19C	161 (3)
C12A—C7A—C3C—C2C	161.9 (14)	C5C—C6C—C19C—O2C	−79 (3)
C12A—C7A—C3C—C4C	−42 (3)	C5C—C6C—C19C—O3C	98 (2)
C13A—C14A—C15A—C16A	0.0	C6C—C1C—C2C—C3C	43.2 (18)
C13A—C1A—C2A—C3A	−159.9 (6)	C19C—O3C—C20C—C21C	174.3 (19)
C13A—C1A—C6A—C5A	173.5 (7)	C20C—O3C—C19C—O2C	5 (4)
C13A—C1A—C6A—C19A	−61.8 (6)	C20C—O3C—C19C—C6C	−172.2 (15)
C14A—C13A—C18A—C17A	0.0	Cl1C—C16C—C17C—C18C	−177.3 (19)
C14A—C13A—C1A—C2A	−104.5 (5)	Cl1B—C16B—C17B—C18B	−178.78 (19)
C14A—C13A—C1A—C6A	136.7 (4)	F1B—C10B—C11B—C12B	178.2 (2)
C14A—C15A—C16A—C17A	0.0	O1B—C5B—C6B—C1B	152.6 (2)
C14A—C15A—C16A—Cl1A	−179.9 (8)	O1B—C5B—C6B—C19B	28.2 (3)
C15A—C16A—C17A—C18A	0.0	C1B—C2B—C3B—C4B	26.2 (3)
C16A—C17A—C18A—C13A	0.0	C1B—C2B—C3B—C7B	−152.95 (18)
C18A—C13A—C14A—C15A	0.0	C1B—C6B—C19B—O2B	120.3 (3)
C18A—C13A—C1A—C2A	82.8 (8)	C1B—C6B—C19B—O3B	−60.9 (2)
C18A—C13A—C1A—C6A	−35.9 (9)	C1B—C13B—C14B—C15B	180.0 (2)
C13C—C14C—C15C—C16C	0.0	C1B—C13B—C18B—C17B	179.8 (2)
C13C—C1C—C2C—C3C	146.2 (15)	C2B—C1B—C6B—C5B	51.7 (2)
C13C—C1C—C6C—C5C	−169.5 (13)	C2B—C1B—C6B—C19B	174.65 (17)
C13C—C1C—C6C—C19C	71.2 (12)	C2B—C1B—C13B—C14B	−94.2 (2)
C14C—C13C—C18C—C17C	0.0	C2B—C1B—C13B—C18B	84.7 (2)
C14C—C13C—C1C—C2C	−35 (2)	C2B—C3B—C4B—C5B	−2.4 (3)
C14C—C13C—C1C—C6C	74 (2)	C2B—C3B—C7B—C8B	−23.3 (3)
C14C—C15C—C16C—C17C	0.0	C2B—C3B—C7B—C12B	153.8 (2)
C14C—C15C—C16C—Cl1C	177.4 (18)	C3B—C4B—C5B—O1B	−177.9 (2)
C15C—C16C—C17C—C18C	0.0	C3B—C4B—C5B—C6B	4.3 (3)
C16C—C17C—C18C—C13C	0.0	C3B—C7B—C8B—C9B	176.9 (2)
C18C—C13C—C14C—C15C	0.0	C3B—C7B—C12B—C11B	−177.1 (2)
C18C—C13C—C1C—C2C	126.7 (11)	C4B—C3B—C7B—C8B	157.5 (2)
C18C—C13C—C1C—C6C	−123.5 (8)	C4B—C3B—C7B—C12B	−25.4 (3)
O1A—C5A—C6A—C1A	141.5 (14)	C4B—C5B—C6B—C1B	−29.6 (3)
O1A—C5A—C6A—C19A	12.7 (18)	C4B—C5B—C6B—C19B	−154.0 (2)
C1A—C13A—C14A—C15A	−174.4 (6)	C5B—C6B—C19B—O2B	−115.2 (3)
C1A—C13A—C18A—C17A	171.6 (9)	C5B—C6B—C19B—O3B	63.6 (2)
C1A—C2A—C3A—C7A	−156.4 (6)	C6B—C1B—C2B—C3B	−50.1 (2)
C1A—C2A—C3A—C4A	10.8 (15)	C6B—C1B—C13B—C14B	141.6 (2)
C1A—C6A—C19A—O2A	130.9 (11)	C6B—C1B—C13B—C18B	−39.5 (3)
C1A—C6A—C19A—O3A	−48.1 (12)	C7B—C3B—C4B—C5B	176.66 (19)
C2A—C1A—C6A—C5A	56.6 (7)	C7B—C8B—C9B—C10B	−0.1 (4)
C2A—C1A—C6A—C19A	−178.6 (5)	C8B—C7B—C12B—C11B	0.1 (3)
C2A—C3A—C4A—C5A	9 (3)	C8B—C9B—C10B—F1B	−178.4 (2)
C3A—C7A—C8A—C9A	−174.9 (5)	C8B—C9B—C10B—C11B	0.6 (4)
C3A—C7A—C12A—C11A	175.8 (5)	C9B—C10B—C11B—C12B	−0.7 (4)
C3A—C7A—C3C—C2C	−86 (5)	C10B—C11B—C12B—C7B	0.4 (3)
C3A—C7A—C3C—C4C	70 (5)	C12B—C7B—C8B—C9B	−0.2 (3)
C3A—C4A—C5A—O1A	−174.7 (17)	C13B—C1B—C2B—C3B	−175.38 (17)
C3A—C4A—C5A—C6A	5 (3)	C13B—C1B—C6B—C5B	176.38 (17)
C4A—C5A—C6A—C1A	−38.6 (16)	C13B—C1B—C6B—C19B	−60.7 (2)
C4A—C5A—C6A—C19A	−167.4 (13)	C13B—C14B—C15B—C16B	0.3 (4)
C5A—C6A—C19A—O2A	−103.6 (14)	C14B—C13B—C18B—C17B	−1.3 (3)
C5A—C6A—C19A—O3A	77.4 (12)	C14B—C15B—C16B—Cl1B	178.56 (18)
C6A—C1A—C2A—C3A	−43.6 (6)	C14B—C15B—C16B—C17B	−1.5 (4)
C19A—O3A—C20A—C21A	86.8 (10)	C15B—C16B—C17B—C18B	1.3 (4)
C20A—O3A—C19A—O2A	4.0 (18)	C16B—C17B—C18B—C13B	0.1 (4)
C20A—O3A—C19A—C6A	−177.1 (7)	C18B—C13B—C14B—C15B	1.0 (3)
Cl1A—C16A—C17A—C18A	179.9 (8)	C19B—O3B—C20B—C21B	−107.9 (4)
O1C—C5C—C6C—C1C	−139 (3)	C20B—O3B—C19B—O2B	−0.8 (4)
O1C—C5C—C6C—C19C	−17 (4)	C20B—O3B—C19B—C6B	−179.6 (2)
C1C—C13C—C14C—C15C	159 (2)		

### Hydrogen-bond geometry (Å, º)

**Table:**

*D*—H···*A*	*D*—H	H···*A*	*D*···*A*	*D*—H···*A*
C8*A*—H8*A*···O1*B*	0.95	2.55	3.478 (3)	167
C2*A*—H2*AA*···O1*B*	0.99	2.44	3.288 (6)	143
C2*A*—H2*AB*···O2*B*^i^	0.99	2.56	3.432 (7)	147
C2*C*—H2*CA*···O1*B*	0.99	2.32	3.278 (18)	164
C2*C*—H2*CB*···O2*B*^i^	0.99	2.42	3.40 (2)	170
C2*B*—H2*BA*···O1*A*^ii^	0.99	2.49	3.315 (8)	140
C2*B*—H2*BA*···O1*C*^ii^	0.99	2.56	3.380 (18)	140
C2*B*—H2*BB*···O2*C*^iii^	0.99	2.58	3.436 (10)	145
C8*B*—H8*B*···O1*A*^ii^	0.95	2.50	3.379 (8)	154
C8*B*—H8*B*···O1*C*^ii^	0.95	2.54	3.417 (19)	154
C9*B*—H9*B*···O2*A*^ii^	0.95	2.54	3.274 (6)	134
C14*B*—H14*B*···O2*A*^iii^	0.95	2.54	3.247 (5)	131

Symmetry codes: (i) −*x*+1, −*y*+1, −*z*+1; (ii) *x*, *y*, *z*+1; (iii) −*x*+2, −*y*+1, −*z*+1.
